# Recruiting and motivating black subjects to complete a lengthy survey in a large cohort study: an exploration of different strategies

**DOI:** 10.1186/1471-2288-14-46

**Published:** 2014-04-03

**Authors:** Patti Herring, Terry Butler, Sonja Hall, Hannelore Bennett, Susanne B Montgomery, Gary Fraser

**Affiliations:** 1 School of Public Health, Health Promotion & Education, Loma Linda University, 24951 North Circle Dr, NH 1511, Loma Linda, CA 92350, USA; 2 School of Public Health, Biostatistics & Epidemiology, Loma Linda University, 24951 North Circle Dr, NH 2005, Loma Linda, CA 92350, USA; 3 Behavioral Health Institute, Loma Linda University, 1686 Barton Rd, Loma Linda, CA 92373, USA; 4 School of Public Health, Loma Linda University, 1709 Nichol Hall, Loma Linda, CA 92350, USA

**Keywords:** Black, African American, Enrollment, Recruitment, Promotion, Re-promotion, Revisit, Multiple strategies, Incentives, Sub-studies

## Abstract

**Background:**

The effectiveness of multiple innovative recruitment strategies for enrolling Black/African American participants to the Adventist Health Study-2 (AHS-2) is described. The study’s focus is diet and breast, prostate and colon cancer.

**Methods:**

Promotions centered on trust, relationship building and incentives for increasing enrollment and questionnaire return rate. Of the sub-studies described, one had a randomized control group, and the others, informal controls. The subjects are from all states of the U.S. and some provinces of Canada. The offer of a Black art piece, follow-up calls, a competitive tournament as well as other strategies accounted for nearly 3,000 additional returns even though they were often used in small subsets.

**Results:**

Flexibility and multiple strategies proved advantageous in gaining the cooperation of Blacks, who are usually reluctant to participate in research studies.

**Conclusions:**

Lessons learned during initial enrollment should help us retain our final Black cohort of 26,000, and obtain new information when required.

## Background

Minorities are under-represented in research studies and enrolling them in dietary cohort studies with long questionnaires, is particularly challenging [1-5] Therefore, it is not surprising that nearly 20 years following the National Institute of Health (NIH) requirement to increase the number of minorities in research studies [6], the response remains very modest. Our experience with Adventist Health Study-2 (AHS-2) and the methods that were tried, contribute to an understanding of this deficiency, but also to ways to help overcome the problem. AHS-2 is one of a relatively small number of contemporary cohorts containing a large number of Black participants. Its major aims are to investigate the associations between dietary soy, dairy, calcium, and long chain fatty acids, and the risk of prostate, colon, and breast cancers. We also aim to compare dietary patterns and other lifestyle risk factors, between racial groups, hoping to explain health disparities across populations. The initial Black recruitment goal was 35,000 returned questionnaires from Black church members and we finally achieved 25,662, a gratifying result as we subsequently estimated that this represented about 30% of the estimated source population, at least as good as that achieved for non-Blacks in AHS-2.

Recruitment consisted of systematically approaching church members throughout the U.S. and Canada to enroll in the study, starting on the west coast and proceeding eastward, then to the South and Canada. This effort lasted for five years (2002–2007). Participants were considered enrolled in the AHS-2 if they completed an enrollment card signifying their desire to participate in the study. They then received a questionnaire from the study headquarters and completed the 50-page self-administered instrument. Once this was mailed to us, they then became official study participants.

Our initial recruitment model fully described elsewhere [7,8], was church-based. Briefly, each of 1003 Black churches were provided and adopted an enrollment goal based on their usual adult attendance. The pastor and a layperson (church recruiter) were selected to represent the study to their congregation and were trained by study investigators, either in person or by telephone. Pastors and church recruiters received a monetary incentive for their promotional efforts, and all Black respondents who returned a questionnaire were mailed a check for $10. The initial strategy included multiple announcements and enrollment requests directed to members who attended church during a 12–16 week period.

We became aware early on however, that although the initial promotional/recruitment model was very effective, that achieving our goal of 35,000 would be challenging. Thus, our team of researchers and local church recruiters proposed a number of other novel ancillary recruitment strategies which we implemented to follow the initial recruitment model as resources allowed. Mostly this was in one region of the country, as the new methods were only being tested rather than implemented generally, and this was usually in a region where the study was promoting or re-promoting questionnaire return at that time. In most of these sub-studies, subjects would have already requested and received a questionnaire, but not yet returned it. Exceptions are the two “Enroll Another” studies (explained later) where subjects had already completed and returned their questionnaires, but they then enrolled new members and encouraged them to return the questionnaires.

In 2004, after nearly three years of recruiting we had covered the West Coast, the Mid-West and much of the East Coast, using the basic promotional/recruitment model plus telephone follow-up (the first ancillary method tested). At this time we launched a re-promotion campaign in an effort to improve the questionnaire return rate from churches where initial promotion was concluded but where goals had not been reached [7]. In addition, successful strategies could be added to the promotional plan in areas not yet reached. During this re-promotion, all pastors’ monetary incentives increased from $350 to $1,000 for large churches (goals taking values greater than 30 completed questionnaires), and to $500 for smaller churches (goals taking values less than 30) and incentives were prorated according to the proportion of the goal achieved. Study-wide, the local church recruiters also received, during re-promotion, an increased prorated monetary incentive (between $100 - $200 depending on church size). As these increases were applied across all churches their effectiveness could not be tested in this study.

In this paper, we evaluate these ancillary recruitment strategies. Although, with one exception [9] the sub-studies described here were not originally designed for formal assessment, it has often proved possible to put together useful control observations, and so estimate the effect of the method to increase enrollment. These observations will also be of interest to other researchers who plan to involve Blacks/African Americans in their studies.

## Methods

The main goal of the additional sub-studies used in this re-promotion was to rapidly increase questionnaire returns. Thus, the timing of the additional strategies, with respect to the main AHS-2 recruitment model, was variable and different strategies started at various times (see Table 1). As it is likely that recruitment at later times was more difficult, given that the remaining target population had not been persuaded by earlier approaches, we group the various approaches by the time of application: a) Sub-studies part of initial promotion; and b) Later re-approach strategies.

a) Sub-studies part of initial promotion

Follow-up telephone calls: Members of 40 Black churches participated in a sub-study to test the effectiveness of follow-up telephone calls made three months after initial promotion. As in most of these sub-studies, the target subjects in these churches were those who had requested questionnaires, but had not returned them. The churches were randomized to either the experimental (received phone calls) or control status in a 2:1 ratio favoring the intervention [5].

Black Art Incentive: In February through April 2004, an innovative incentive strategy included the promise of a free Black art poster to those who returned their questionnaires [9]. This pilot study was promoted especially during February 2004, Black History month, as celebration activities were already in place at most of the participating churches. The choice of two posters was displayed on the back page of the questionnaire, and members were encouraged to select one as a “thank you” if they returned their questionnaire within six weeks. In the first of such pilots, members had a choice of either a poster or the usual $10. In a second pilot, held soon after in different churches but in the same part of the country, members received both an art piece and $10 for completing and returning their questionnaire promptly.

b) Later Re-approach strategies

Operation Mission Possible 30,000: From April until September 2005, with 37,000 surveys distributed to Black members 19,000 had not returned their questionnaires. Thus, we launched “Operation Mission Possible 30,000” to both Black and non-Black potential study members who had received a questionnaire at an earlier time. Operation 30,000 was developed with the intention of encouraging a total of 30,000 questionnaires returns and of that 20,000 of these we hoped would come from Black subjects.

During this time we attempted to call 615 church recruiters; unfortunately we were not able to reach all 615. For those churches whose recruiters we could not reach by phone we contacted their pastors. We reached nearly half (253) and sent 209 packets to the churches, containing 7 simple strategies they could use to get their members to complete their questionnaires and return them. Some of the suggested strategies included a tag team approach whereby one member completes his questionnaire and then finds and encourages another member to also complete his/her questionnaire. Church members continue to tag each other this way until many members complete the questionnaires. We also encouraged the buddy system whereby enrolled church members paired up with another church member to complete their questionnaires together, thereby motivating each other to not give up until the questionnaire was completed and mailed.

This strategy/method was instituted in all regions of the U.S. except Allegheny East (New Jersey, Delaware and contiguous eastern portions of Pennsylvania, Maryland, and Virginia), Florida, and South Central (southeastern states including Alabama, Mississippi, Tennessee) where initial promotion was within six months or had not yet begun.

**Table 1:** sequence and location of the sub studies recruitment activities 2002 - 2006

	**West Coast**	**Mid-America**	**Great Lakes**	**North East**	**Allegheny East**	**South/Florida**	**South West**
Initial promotion	6/2002 – 7/2003	5/2003 – 12/2003	01/2004- 6/2004	5/2004 – 12/2004	05/2004 – 12/2004	7/2004 – 6/2005	07/2005- 6/2006
Telephone follow-up	1/2003 – 6/2003						
Black Art						06/2003- 12/2003	
Operation 30,000	5/2005 – 10/2005	5/2005 – 10/2005	5/2005 – 10/2005	5/2005 – 10/2005			
Student recruiters*	12/2002 –10/2005					6/2004 – 9/2004	
Local recruiters	04/2006 -10/2006	04/2006 -10/2006					
Tournament of Healing					05/2006 – 08/2006		
Lake Region $20			05/2006 -08/2006				
$2/$10**	1/2006 – 4/2006	1/2006 – 4/2006	1/2006 – 4/2006	1/2006 – 4/2006	1/2006 – 4/2006	1/2006 – 4/2006	1/2006 – 4/2006

*Of the 9 matched sets of 5 churches, one on the West Coast overlapped with *Operation 30,000* (in this set intervention did no better than controls). *Operation 30,000* will have affected intervention and control churches equally and should have caused no bias.

**The apparent overlap in time between the $2/$10 sub-study with Local recruiters is only of trivial magnitude as the $2/$10 was not church- but individual subject-based. It affected only 1000 randomly selected subjects out of 80,000 enrolled, and affected equally both control and intervention churches of other sub-studies.

### Student recruiters

Between 2002 and 2006, fifteen students from Oakwood College/University, Huntsville, Alabama and Loma Linda University, Southern California (Adventist tertiary educational institutions) visited 23 churches in California, New York (not in analytic data due to difficulty finding appropriate control churches), Florida, and Tennessee and made presentations and phone calls to encourage questionnaire returns. Students also promoted AHS-2 at their home churches when on school breaks. Some students attended large national church conferences, regional camp meetings and university alumni weekends, where they manned AHS-2 recruitment booths. Students received $20 for each returned questionnaire that was labeled with their assigned bar code number.

### Local recruiters Re-promotion

In some cases, this local recruiter was the original lay church recruiter from the basic recruitment model, but in most cases it was someone new. Pastors were not directly involved, but if the extra efforts helped the church to reach its goal, this increased the pastors’ monetary incentives. In most churches, local recruiters individually contacted members who had received a questionnaire, encouraged questionnaire completion and conducted group sessions to assist church members with completing their questionnaires. The local recruiter received $10 for each of the first five returned questionnaires and $20 for each returned after the first five.

### Tournament of healing

Allegheny East Conference was selected for a pilot program that involved a spirited competition among churches which had completed enrollment one to two years previously. Churches were divided to three categories (small, medium, and large) and within each category churches competed for the most questionnaire returns. The winning churches were promised Bibles, engraved with the church’s name. The two criteria for selecting the competing churches were that the initial return rate was below 50% of the church’s goal, and that the pastors gave consent. Nine small, 13 medium-size, and 16 large churches were selected to participate. As a motivational tool, each church, and only that church was able to “heal” a specific cold symptom of a hypothetical “very sick clerk” at the AHS-2 headquarters, by achieving their church enrollment goal. We mailed to each church a chart of symptoms as goal points for healing. Local church recruiters were the main motivators over a period of three months.

### Operation lake region

In 80 churches of the Lake Region Conference (Wisconsin, Illinois, Indiana, and Michigan) we tested whether doubling the church members’ monetary incentive from $10 to $20 would improve questionnaire returns. First our research assistants telephoned all 51 pastors, hoping for their support, and explaining this last chance to achieve their church goal so maximizing their monetary incentive. If the pastor could not be contacted by phone, they were sent a certified letter requiring a signature of receipt. Then, all 1,614 members in the 80 churches who had not returned their questionnaire were sent a letter explaining that their monetary thank you gift would double from $10 to $20 upon the return of their questionnaire.

### Incentives to enroll another member: $2 immediately or $10 late

These sub-studies tested the willingness and ability of church members who had already returned their questionnaires, to use their enthusiasm to persuade others who had not completed their questionnaire to do so. Two different monetary incentives were tested for the enrollers, who were already members of AHS-2.

For the *$2 “up-front” incentive* we randomly selected 500 potential enrollers who had already returned a questionnaire, and sent them a brand-new, crisp $2 bill, a thank you letter, and a questionnaire to give to a friend, church member, or family member to complete. For the *$10 “after questionnaire return” incentive,* another randomly selected 500 participating church members were sent the same request, but were promised that they would receive a $10 incentive upon our receipt of the enclosed questionnaire completed by a newly enrolled member.

#### Statistical methods and selection of controls

Churches received individual goals for the number of returned questionnaires. Membership rosters were often inaccurate so these goals were determined as 50% of the number of bulletins printed for the weekly church services, as this number approximately matched the number who regularly attended at that time.

The measure of effect used for all sub-studies is the proportional increment, defined as (increment in returns during the sub-study intervention period)/Goal. The analyses for the two “Enroll Another” sub-studies are described separately below. The other sub-studies used church as the unit of observation and analysis. Details of control selection for the telephone follow-up and art poster sub-studies have been published [5,9]. However, these whole studies were published in more details in the previous publications than they are explained here. We describe all study endeavors in this one paper so that readers can understand the context of each sub-study and make informed comparisons. Thus the design and results of these two studies are also summarized here. More details are found in the original reports.

With the large number of churches involved, we were able to find suitable control churches to allow a useful comparison, where necessary. Intervention and control periods were always defined as the 6 months after the intervention or defined control status began, and questionnaires that were returned in that period from churches or members, were counted. It was also required that these churches had not participated in other special promotions in the 6 month period before the study of interest began, to avoid contamination from other interventions.

Controls were of two main types: a) churches acted as their own controls during an adjacent 6 month time period when there was no intervention; or b) Other churches that were matched by size, geographical region and time period, acted as controls. In the first situation we were aware that the underlying rate of return of questionnaires in a control period was gradually diminishing as time passed after the main enrollment promotion in a particular region. Thus a control period that followed the intervention period could give a non-conservative bias. Thus we either: a) took control periods for some churches before and other churches after the intervention period. Where the earlier and later periods involved different numbers of churches, the likelihood for the later control periods was weighted by the ratio a/b where a is the number of churches in the early and b in the later periods, as a means of restoring balance; b) took the average for each church of two control periods, that immediately before and immediately after the intervention period, where there were no other interventions in these control periods.

The model used for statistical analysis, for all except the two “Enroll Another” sub-studies, assumed an over-dispersed Poisson process governing the returns from churches. The returns were evaluated during intervention and control periods as a proportion of the predetermined goals given to each of the J churches. The Poisson parameter, λ (the increment in returns), was modeled as $\lambda_{j}=G_{j}\;\exp\;\left(\alpha +\beta_{1}I_{j}+\sum_{j=1}^{J}\left(\beta_{2j}C_{j}\right)+e_{j}\right)$ where G is the goal for church j, I is intervention status (0 = control; 1 = intervention), the C_j_ are 0/1 variables indicating, in different sub-studies, either church identity or group identity (of a matched set of churches), and e is a Poisson error. Thus λ_j_/G_j_ is the proportional increment (PI) predicted by the model, and β_**1**_ is the coefficient of interest that indicates whether the intervention made a detectable difference. A p value tests the null hypothesis that β_**1**_ = 0, which is equivalent to testing that E (PI_I_) = E (PI_C_), where subscripts I and C refer to intervention and control. The β_**2j**_ coefficients are used, where necessary, to extract a component of variance due to individual churches or matched groups of churches, in a repeated measures approach. Coefficients were estimated by maximizing the likelihood, programming in the R language. An over-dispersion parameter was calculated [10] and used to adjust the z score used to test hypotheses. This accounts for the fact that churches are not identical units (aside from random error), even conditional on covariates (where these were included).

The two “Enroll Another” sub-studies considered the 500 selected members as the units of observation, with the understanding that any returns from the additional questionnaires that they were each supplied was an increment that could only result from the intervention. Thus, there are no control groups. The statistic of interest is (number of returns during the sub-study)/750 and its 95% confidence interval, treated as a proportional increment. At the time of these sub-studies we were at about 2/3 of the study goal for the whole cohort. So if the interventions had been applied to all current respondents in the cohort, the denominator of the effect statistic (i.e. the goal) would have been 50% larger than the number of current participants. We proportionately apply the same reasoning to the 500 subjects actually in these sub-studies, so providing a value for G and a metric that can be compared to PI values from other sub-studies that were based on church as the unit of observation. Equivalently one could think of these 500 subjects as a synthetic church that was at 2/3 of its goal of 750 returns. The observations are treated as 500 independent Bernoulli outcomes, and the analyses use logistic models having only a constant term. The resulting predicted proportions of returns were multiplied by 2/3 to change the denominator to the sub-study goal. The alpha coefficient, its standard error, and the confidence interval for PI_I_ were all also adjusted appropriately.

Table 2 summarizes the control selection and analytic strategies for each study.

**Table 2:** Details of control selection for each sub-study

**Sub study**		**Type of control**	**Number of controls/Intervention units**	**Timing of control period compared to intervention**
A. Associated with baseline promotion				
	Black Art Alone compared to $10	Other churches*	24/19	Concurrent
	Black Art + $10 compared to $10		24/62	Concurrent
B. Early post-baseline				
	Telephone follow-up	Other churches (randomized)	13/27	Concurrent
C. Mid post-baseline				
	Operation 30,000	Self (152 churches)	N/A	71 churches before and 81 after intervention period^§^
	Student recruiters	Other matched churches	9/36	Concurrent
	Local Recruiters	Other matched churches	12/10	Concurrent
	Tournament of Healing	Other matched churches	22/38	Concurrent
D. Late post-baseline				
	Lake Union $20	Self (80 churches)	N/A	All controls are an average of periods before and after intervention
	Enroll Another $2 bill^†^	None	0/500^†^	N/A
	Enroll Another $10^†^	None	0/500^†^	N/A

*Adjusted also for church size in the statistical model.

^†^These studies are individual subject, rather than church-based.

^§^Likelihood weighted to restore balance.

### Ethics statement

This research was performed with the approval of the Research Affairs Committee of Loma Linda University in compliance with the Helsinki Declaration.

## Results

Table 3 shows the results from the various sub-studies. The studies are divided into time periods ranging from the “being part of the initial promotion” to “9-36 months following initial promotion”, as it is clear that the challenge of promoting questionnaire returns becomes harder as time passes. Non-participating subjects have already rejected previous approaches.

**Table 3:** Results of ancillary strategies to motivate enrolment AHS-2 among black adventists: proportional increments (PI)* due to the interventions

**Sub-study**	**Beta coefficients (SE)**	**p**^**+**^	**E (PI**_**I**_**)**	**E (PI**_**c**_**)**	**E (PI**_**I**_**)-E (PI**_**c**_**)**
A. Association with Baseline promotion					
Black art alone compared to $10^§^	0.049 (0.156)	0.754	0.463	0.441	0.022
Black art plus $10 compared to $10^§^	0.512 (0.237)	0.033	0.737	0.441	0.296
B. Early post-baseline (3 months)					
Telephone follow-up	1.043 (0.337)	0.004	0.098	0.035	0.063
C. Late post-baseline (9–36 months)					
Student recruiters	1.002 (0.353)	0.008	0.041	0.015	0.026
Local recruiters	1.988 (0.639)	0.007	0.033	0.005	0.028
Operation 30,000	0.152 (0.120)	0.207	0.035	0.030	0.005
Tournament of healing	1.345 (0.312)	0.0001	0.054	0.014	0.040
Lake Union $20	0.558 (0.145)	0.0002	0.032	0.018	0.014
	Alpha coefficient (SE)		E (PI_I_)	95% CI	
Enroll another $2	-2.861 (0.198)		0.036	(0.025, 0.052)	
Enroll another $10	-3.168 (0.228)		0.027	(0.017, 0.041)	

*Increment expressed as proportion of the goal; PI = (returns during stipulated period)/Goal. PI_c_ is PI during the control period and PI_I_ is PI during the intervention period.

^+^p tests the null hypothesis that the beta coefficient related to PI_I_/PI_c,_, equals zero, and similarly tests that PI_I_ = PI_c._

^§^Approximate mean values used for covariates in the statistical model (reference #9).

The first, which was the most effective sub-study, was the use of Black art posters [9] as part of the initial promotion. When compared to $10 alone, the combination Black art plus $10 appeared to increase the proportional increment by 0.296, whereas Black art alone was not clearly better than the $10 incentive.

The second sub-study used follow-up phone calls immediately after the initial promotion and involved most regions of the country. This resulted in nearly 800 additional questionnaire returns, and a difference in proportional increments of 0.063. These were in effect late returns to the initial promotion prompted by the phone calls. We found no advantage to such calling in a similar study among Whites [5].

Nine to 36 months after initial promotion at mid- to late-post-baseline, five other controlled sub-studies typically found that during the control periods, returns as a proportion of goal during a 6 month study period were very low (around 1-3%). Focusing on the difference between E (PI_I_) and E (PI_C_) as the measure of effect, *Operation 30,000* appears to be the least effective and the effect was not statistically significant. All other controlled sub-studies had a statistically significant effect. The magnitude of the significant effects ranged between 0.014 to 0.040. These modest, but apparently real effects, if they had been applied to the whole study independently would thus have individually produced 490 to 1400 additional returned questionnaires. The *Tournament of Healing*, a competition, appeared to be the most successful.

The two *“Enroll Another*” studies did not need a control group as the control status of zero returns was reasonably assumed. They involved subjects who had already returned a questionnaire as the promoters, and occurred relatively late in the post-baseline period. In response to the *Enroll Another $2 and $10* monetary sub-studies, there were 0.036 (95% confidence interval 0.025-0.052) and 0.027 (95% confidence interval 0.017-0.041) returns as a proportion of goal. Thus, these very late post-baseline strategies, that often may have followed previous strategies in some churches, had small effects that if applied to all cohort members who had already returned a questionnaire, would have been predicted to produce 1260 and 945 returns, respectively. However, in this case the *$2 “Up-Front*” would have been very expensive, around $37 per returned questionnaire.

## Discussion

We believe that collectively these sub-studies/methods resulted in the return of nearly 3,000 additional questionnaires, even though they were often applied only to small sub-samples and at different times post-baseline. Clearly some approaches were more successful than others, though this was greatly dependent on timing after initial promotion. Some of these strategies were also applied in churches aside than those used for these analyses. These other churches were excluded from analyses because either more than one strategy was employed within the same time period, or we could not find a suitable control church or control period for them (see Table 1 for timing of sub-studies and any overlap).

### The most successful strategies

The difference in proportional increments was greatest for the *Black Art project* as an enhancement of the initial promotion strategy [9]. We selected Black art as a culturally appropriate, appealing, and meaningful incentive to the target population, and its success is consistent with other findings [2-4]. The idea of using Black art had a very positive reception from church recruiters at an earlier training session, and we decided to test it mainly during February (Black History month). It thus seems possible that enrollment could have been 67% higher (30% of goal), given the time and resources to implement this strategy across the cohort.

The *Follow-up Phone Calls* sub-study [5] produced a higher incremental questionnaire response rate among Black participants relative to controls, when applied three months after the initial promotion had begun. Those participants with whom we actually spoke were more likely to return their questionnaire than those who received only a telephone voice message. This is consistent with other data showing that Black subjects are particularly responsive to personal appeals [8,11]. Although effective, these calls were moderately costly in terms of manpower and telephone charges, we did judge them to be cost-effective. As this sub-study was conducted early in the recruitment years, we continued to use follow-up phone calls (throughout initial promotion/recruitment and re-promotion) in all the Black churches in order to maximize response. Finally we believe that they resulted in more than 2,000 additional members returning their questionnaires.

The *Tournament of Healing* proved quite successful in motivating members to return questionnaires. Our plan was to capitalize on the proven motivational nature of competitions [12] to improve questionnaire returns. As anticipated it reenergized church members in some (but not all) churches, and again raised enthusiasm for the study.

### Less successful strategies

The less successful strategies were generally those used during re-promotion 2–3 years post-baseline, but still may if employed alone, have each produced an extra 1,000 returns (around 3% of goal). An exception is *Operation 30,000* that did not appear to be effective. After three years of recruiting/promoting and re-promoting we believed that church members and pastors had become desensitized to hearing about the study and became less inclined to complete and return their questionnaires.

However, if these strategies had been inexpensive, they may still have been worthwhile, resulting in 200–600 additional completed questionnaires. However, in our view the costs of applying any of these to the whole cohort would not be justified by the likely response. Many of the sub-studies were conducted at a time when we were well through the recruitment period so their widespread implementation was not an issue unless effectiveness had been outstanding. The student and local recruiter strategies are relatively expensive as many recruiters need to be trained in order to cover hundreds of churches. Offering $20 per returned questionnaire would cost only $10 extra per questionnaire, but appeared to have only a small effect.

### The cost effectiveness of the strategies

We did not collect data that allow detailed cost-effectiveness analysis. However, it is clear that the *Black Art* project at relatively little cost prompted an outstanding response. The *Telephone Follow-Up* and *Tournament of Healing* also delivered modest but worthwhile responses, again at a manageable cost. Responses in other sub-studies were lower. It should be noted that a 1% increment after the conclusion of the initial promotion would amount to about 230 additional questionnaires.

The “*Enroll Another*” strategies would become expensive when applied to the whole cohort as they require a questionnaire to be mailed to all current respondents, for only a small proportionate return. We do not know whether some of these results would have been different if offered early in the recruitment strategy or even initially.

Various types of controls were used in the different sub-studies that were dictated by the timing and flow of events in the main cohort study. While randomized controls are usually the best option, using the same church as its own control should also control many confounding factors. Even when other churches, matched as possible, were used, the over-dispersed Poisson analyses should largely compensate for other non-systematic differences between intervention and control churches.

### The use of multiple strategies

Since Blacks in general are reluctant to participate in research for multiple reasons [2-4,8], it is logical to propose that multiple strategies with emphasis on flexibility would be advantageous to gain the cooperation of a large sample of Black participants [1,13]. In support of this Williams and associates [14] found an advantage to using multiple strategies, as some recruitment methods were effective in one community, but not in others; thus they found it necessary to make changes quickly, and implement new methods when the standard methods lost effectiveness, as did we.

Others reported that in order to overcome the difficulties and barriers in recruiting Blacks to research studies, not only were multiple strategies needed [4,8,15-17], but appropriate incentives were imperative, noting that a lack of incentives may actually hinder Blacks from participating. Dickert and Grady [18] also discovered the motivating value of monetary incentives. Previous focus group results [4] in Adventists had also clearly indicated that they were likely to be important. It was interesting that we find that doubling the incentive from $10 to $20 late in the recruitment period had only a small effect.

## Conclusion

As expected, recruitment methods used early proved more successful as compared to those used later in promotions. An apparently highly effective additional incentive during the initial promotion (the Black art) emphasized cultural sensitivity [4,8,13,17,19] rather than additional cash. The successful telephone follow-up early after the initial promotion emphasized the personal contact [8,11]. A somewhat later, but successful strategy, emphasized competition between churches for a reward tailored to the competitors (Bibles for the winning church). Other strategies were less successful, although it is possible that for some it was because they were employed long after the initial promotion. Nevertheless, most strategies appeared to increase response in a modest way.

These results suggest to us that if we were to promote again in this population we would, in addition to using the successful initial promotion strategy [8]: a) Add the Black Art offer to the initial promotion; b) Add the follow-up phone call to encourage the slower respondents to respond to the initial promotion strategy; c) Consider the use of competitions between groups of churches, perhaps as part of the initial strategy (although tested here as a later strategy).

We hope that others interested in recruiting Blacks to research studies can learn from our experiences and build on the lessons learned from this study. More formal and randomized controls in future work, perhaps particularly directed toward the Black Art strategy, would provide even stronger results. Further research is needed to examine whether our findings apply to the recruitment of Blacks in general, and to church congregations of other denominations.

## Competing interests

The authors declare that they have no competing interests.

## Authors’ contributions

PH participated in the collection of the data, concept and design, drafted the manuscript, and provided general supervision of the research group. TB participated in the collection of the data and edited the manuscript. SH participated in the collection of data and edited the manuscript. HB participated in over-all management of the research project, data collection and analysis. SM participated in the editing of the manuscript. GF participated in the concept and design of the manuscript, analysis, interpretation of the data, and editing of the manuscript. All authors gave final approval of the version to be published.

## Pre-publication history

The pre-publication history for this paper can be accessed here:

http://www.biomedcentral.com/1471-2288/14/46/prepub
